# Darning by Machinery

**Published:** 1875-09

**Authors:** 


At last woman is to be emancipated from a kind of slavery which has been endured for centuries, and from which there has been deemed to be no escape. Henceforth, the trying labor of mending stockings and other knit or loosely woven goods, is to be done by machinery. A Mr. O. S. Hosmer of Boston, has invented a machine which does the work to absolute perfection, and does it very quickly, alike on fine and on coarse goods. We have operated the machine amd can testify to its good qualities. An expert operator can darn a good sized hole—say an inch or two in diameter, in one minute. We saw half a dozen holes darned, and were then invited to try our hand at the business. We selected a pair of thin drawers having a hole about three-quarters of an inch across, and badly worn all around the hole. It took us just two and one half minutes to mend the hole. At the same time and by the same operation, the thin parts around were reinforced and made strong. We venture to say that the garment will wear through anywhere else but in that darn, so thoroughly does the machine do its work.

The machine sells for $10, which seems low. To look at it, one would suppose that this price would not re-imburse the cost of manufacture. It looks as costly as a sewing machine, excepting that it has no table—a clamp accompanying it, by which it is quickly attached to any table at hand.

It will be admitted by all that this machine meets a very general want. By its aid, all the darning which a dozen persons would need, can be done in twenty or thirty minutes weekly. Moreover, anybody can work the machine, and only the most barbarous treatment would put it out of order. Darning will no longer be "mother's work," exclusively, but the boys and girls of the family will vie with one another as to which shall have the privilege of operating this fascinating little invention.

This device is new, and is even now scarcely ready for the market. It has taken Mr. Hosmer ten years of study and experiment to make this machine a perfect one. At last he has brought it to perfection, and a large number are being made. During the present month, all orders will be filled.

Mrs. H. S. Hutchinson, the well-known manager of the "Dress Reform Committee," is the agent for the sale of these machines, and will be prepared to supply them at her rooms, No. 16 West 14th street, New York City. She also exhibits the machine at the fair of the American Institute, in the autumn of 1875. In the same connection she presents the servicable under-garments for women and children, which the Dress Reform Committee have introduced so widely, and which are so rapidly growing in public appreciation and favor.

### COOKS THAT KILL.

Somebody has said that "women are natural cooks." If this is true of women as a class, it is, at least, sadly untrue of women upon whom the average New-Yorker has to depend for the food upon which he strives to exist. These are botches, failures. We have been compelled, for many months, by the exigences of life, to eat beef-steak which the old Irish cook of a pretentious hotel here, had covered with warm water after the broiling process was ended! This was her regular system, and no expostulation would induce a change. The object was, as she explained, to make a lot of "nice water-gravy!" Of course her steak, with the most of the savory and valuable juices washed out of it, was about as palatable and nourishing as a damp rag. The meat was bought for the best and paid for at the highest rates, but it was ruined—utterly and absolutely—by the dire stupidity of an ignorant creature, who assumed to be a "cook" because she knew enough to expose food to the influences of heat, and to remove it from those influences before it was entirely burned up.

The fault, in this case, was with the person in whose charge this cooking automaton was supposed to be. The mistress was ignorant, and so the servant saw that her own ignorance had just as high a commercial value as skill. She was punctual; she never failed to have her ruined meat, her under-done vegetables, and her soggy, sour bread, on hand in time. So she held her place for years and made several thousand persons dyspeptics for life.

Why are we such stupid people? Why do we submit to the grossest of impositions—the imposition of misery and disease and wastefulness—on the part of those whom we pay to prepare our food.

When a man undertakes to build for himself a house, it is the general rule that he exercises the closest care that every portion of the structure shall be, in design and material, the best. He employs a capable architect, a thorough builder, selects stone, brick, mortar, and other components of his fabric, with a rigid scrutiny which leaves no doubt in his mind but that his dwelling will be a strong and lasting shelter. Then he decorates, furnishes, searches for ingenious devices of household convenience, and finally enters his new habitation secure in his belief of its excellence. Is it not strange that all his labor is done for a roof which may cover its owner only until to-morrow; for a home which the vicissitudes of fortune may wrest from him in a day, or which of his own choice he may abandon before the mortar is perfectly dry; while to the structure in which Providence has ordained he shall exist for a lifetime,—his body—but secondary consideration is given?

The trouble lies deep. We have no educated cooks. Our daughters are not taught how to cook accurately, as the chemist in the laboratory teaches his pupils. Our fashionable American society deems it altogether vulgar, if not quite dishonorable, to engage in household avocations. Thousands of families in New York, to-day, are wasting good food by bad cooking, while intelligent girls are barely keeping the breath of life in them, in shops and factories.

